# Biomechanical Effect of Hybrid Dynamic Stabilization Implant on the Segmental Motion and Intradiscal Pressure in Human Lumbar Spine

**DOI:** 10.3390/bioengineering10010031

**Published:** 2022-12-26

**Authors:** Chih-Kun Hsiao, Yi-Jung Tsai, Cheng-Yo Yen, Yi-Chen Li, Hao-Yuan Hsiao, Yuan-Kun Tu

**Affiliations:** 1 Department of Medical Research, E-Da Hospital, Kaohsiung 82445, Taiwan; 2 Department of Orthopaedics, E-Da Hospital, Kaohsiung 82445, Taiwan

**Keywords:** dynamic stabilization systems, Dynesys-Transition-Optima, range of motion, intradiscal pressure

## Abstract

The hybrid dynamic stabilization system, Dynesys-Transition-Optima, represents a novel pedicle-based construct for the treatment of lumbar degenerative disease. The theoretical advantage of this system is to stabilize the treated segment and preserve the range of motion within the adjacent segment while potentially decreasing the risk of adjacent segment disease following lumbar arthrodesis. Satisfactory short-term outcomes were previously demonstrated in the Dynesys-Transition-Optima system. However, long-term follow-up reported accelerated degeneration of adjacent segments and segmental instability above the fusion level. This study investigated the biomechanical effects of the Dynesys-Transition-Optima system on segment motion and intradiscal pressure at adjacent and implanted levels. Segmental range of motion and intradiscal pressure were evaluated under the conditions of the intact spine, with a static fixator at L4–5, and implanted with DTO at L3–4 (Dynesys fixator) and L4–5 (static fixator) by applying the loading conditions of flexion/extension (±7.5 Nm) and lateral bending (±7.5 Nm), with/without a follower preload of 500 N. Our results showed that the hybrid Dynesys-Transition-Optima system can significantly reduce the ROM at the fusion level (L4–L5), whereas the range of motion at the adjacent level (L3–4) significantly increased. The increase in physiological loading could be an important factor in the increment of IDP at the intervertebral discs at the lumbar spine. The Dynesys-Transition-Optima system can preserve the mobility of the stabilized segments with a lesser range of motion on the transition segment; it may help to prevent the occurrence of adjacent segment degeneration. However, the current study cannot cover all the issues of adjacent segmental diseases. Future investigations of large-scale and long-term follow-ups are needed.

## 1. Introduction

Many surgical techniques for lumbar degenerative diseases have been developed, among which decompression and fusion surgery (such as posterior lumbar interbody fusion, PLIF) is one of the most used therapies and is considered the gold-standard procedure. Despite the many benefits of fusion surgery, there are several complications associated with this technique, including instrumentation failure, adjacent segment degeneration, and pseudoarthrosis [1,2,3,4,5,6,7]. Biomechanical studies also showed increased segmental motion, intradiscal pressure (IDP), shear loading, and changed contact patterns within the adjacent levels [8,9,10,11]. To solve these problems, the non-fusion (dynamic stabilization) concept was introduced to address the adverse effect of traditional fusion.

Dynamic stabilization describes the treatment method of achieving stabilization by maintaining the disc with a controlled motion of the segment [12,13,14]. It reduces the risk of accelerated degeneration at adjacent levels, which is the major concern in fusion. To date, various posterior dynamic stabilization systems have been developed and reported in the literature that can be categorized as *posterior interspinous spacers* and *pedicle screw-based* systems.

The first lumbar interspinous process decompression device, X-STOP (St Francis Medical Technologies, Alameda, CA, USA), was introduced in the US for the treatment of patients with neurogenic intermittent claudication due to spinal stenosis. The X-STOP is designed to limit extension movement at the individual stenotic level [15,16]. The Coflex device (Paradigm Spine, LCC, New York City, NY, USA) was first introduced by the French orthopedic surgeon Jacques Samani as an alternative to arthrodesis. The Coflex device is designed to release the facet joint loading, restore the foraminal height, and provide stability to improve the clinical outcome of surgery [17]. The DIAM implant (Medtronic, Memphis, TN, USA) system is designed with a silicon core and a polyethylene cover. Three mesh bands are used to secure the implant: two of them are around each spinous process and the other around the supraspinous ligament [18]. Boody et al. [19] compared the efficacy of the DIAM spinal stabilization system compared with nonoperative treatment for patients with lower-back pain and lower-lumbar-disc degenerative disease. Their results showed that the DIAM device demonstrates improvement in back pain scores maintained to a 2 year follow-up timepoint and performed superior to conventional nonoperative treatment regimens commonly used in lower-back pain.

The pedicle screw-based dynamic stabilization system aims to reduce the stiffness of the instrumentation to allow for more physiologic load transmission at the instrumented levels. Various design concepts, such as more flexible, smaller-diameter metallic rods, hinged pedicle screw heads that allow motion, damper components in the longitudinal elements, and more flexible rods made of nonmetallic biomaterials, have been introduced to achieve this goal [20]. In 1994, the semi-rigid dynamic stabilization system and dynamic neutralization system (Dynesys, Zimmer CH) were developed as an alternative rigid lumbar fusion using the pedicle system and became one of the most popular systems available [21,22,23]. Although some short- and mid-term follow-up studies showed good results [24,25,26,27,28], the retrospective cohort study showed no significant difference between dynamic and rigid stabilization of the lumbar spine for patients with degenerative disc disease [29]. Several studies also reported contradictory results, indicating that Dynesys dynamic stabilization system may not have a significant advantage in clinical measurements, motion preservation, and adjacent disc protection, with the revision surgery rates as high as 30% [26,28,29,30,31,32,33].

To limit stress concentration at the level above or below the fusion and prevent the development of accelerated degeneration at the adjacent segments, a hybrid device, Dynesys-Transition-Optima (DTO) (Zimmer Spine), which consists of two components, dynamic (flexible and non-fusion) and static (rigid and fusion), was developed [34,35]. In the hybrid application, a fusion is performed at the injured or degenerated segment using rigid implants applied posteriorly, with dynamic stabilization extended to levels above or below the fused segments. Some in vitro and biomechanical studies showed that the DTO system can limit flexibility through a polyethylene terephthalate cord and polycarbonate urethane spacer. A 2 year follow-up showed that patients with spondylolisthesis and those with stenosis showed improvements and similar disability and pain scores after surgery using the DTO system. The lumbar alignment was also well maintained [36,37]. A study over 5 years indicated that segment-by-segment treatment with “Dynesys hybrid stabilization” combined with interbody fusion is technically feasible, safe, and effective for the surgical treatment of multilevel degenerative disc disease [33]. Maserati et al. followed 24 patients who underwent DTO stabilization and fusion for a midterm period, and their results demonstrated satisfactory outcomes with significant improvements [25]. Therefore, it may serve as an alternative to multilevel arthrodesis. Implantation of a motion-preserving dynamic stabilization device immediately adjacent to a fused level instead of extending a rigid construct may reduce the subsequent development of adjacent segment disease [24].

Although DTO allows the dynamic stabilization to be performed in the adjacent level to fusion, the medium-term results showed satisfactory outcomes with significant improvements. Some studies also reported on the clinical experience with the DTO hybrid system, but clinical outcomes in long-term follow-up still need to be determined [31,32,37,38]. In addition, more information is needed on the biomechanical behaviors of hybrid dynamic instrumentation and their application, as well as convincing evidence that the DTO system provides biomechanical benefits to patients. Therefore, this study aims to biomechanically investigate the effects of hybrid stabilization (DTO) on the kinematic motion and IDP at the adjacent levels of the fusion using a hybrid sequence test protocol.

## 2. Materials and Methods

### 2.1. Specimen Preparation

Six intact cadaveric human spine specimens from T-11 to the sacrum with a mean age of 68.5 years (SD = 18.6, range 60–78 years; four males and three females) were tested at our laboratory. The institutional review board approved the usage of cadaver specimens in this study in E-Da Hospital (No. EMRP105-042). All specimens were stored in double-sealed bags and maintained at −30 °C when not in use. Before the experiments, each specimen was gradually warmed to room temperature (20 ± 3 °C) until fully thawed (about 12 h). Saline-soaked gauze was wrapped around the specimen to prevent dehydration. To prepare each specimen for testing, all muscular tissues were removed whereas ligaments, joint capsules, and discs were kept intact. Before testing, a quantitative computed tomography (CT) scan (Light Speed VCT, GE Medical Systems, Chicago, IL, USA) was carried out to determine the bone mineral density (BMD) for each specimen. The relative values of BMD were evaluated using two known density calibration phantoms (160 and 320 mg/cm^3^) scanned simultaneously with the specimens. The Hounsfield units (HU) scale value was used to determine the bone density of the vertebrae. The mean measured trabecular BMD of the instrumented vertebrae for all specimens was 215.6 mg/cm^3^ (SD = 32.8).

### 2.2. Description of Hybrid Dynamic Stabilization System and Surgical Treatment

**Figure 1:**
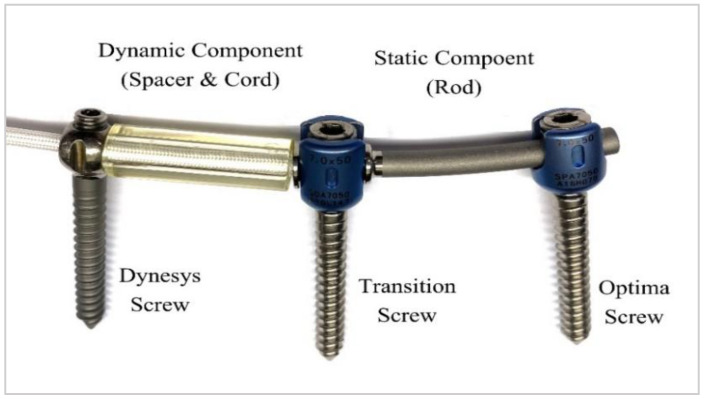
Photograph of the Dynesys-Transition-Optima (DTO) (Zimmer Spine) implant, which is a hybrid (cord–rod) construct comprising a polyester spacer (outer diameter = 11.0 mm and thickness = 2.0 mm) and cord at the dynamically stabilized segment (dynamic component), a titanium rod (diameter = 6 mm) at the rigid fixated segment (static component), and a transition screw connecting the static and dynamic components. The length of the DTO spacer and static components is determined on the basis of the native distance of the pedicles.

The Dynesys Transition Optima (DTO) device (Zimmer-Biomet Spine, Denver, CO, USA) is a unique pedicle screw/rod-based dynamic stabilization system that combines the well-known Dynesys dynamic stabilization system and the OPTIMA ZS spinal system (Figure 1). This device includes two major components: the dynamic (called Dynesys fixator) and the static (called static fixator or fusion) components. The dynamic component comprises a combined polyethene terephthalate (PET) tension cord (100 mm in length and 4.5 mm in diameter) and a preloaded polycarbonate urethane (PCU) flexible tube spacers with an outer diameter of 11.0 mm and tube thickness of 2.0 mm. The static (fusion) component consists of a titanium rod (6.0 mm in diameter) and a transition screw connecting the fusion and dynamic segments. The DTO system allows for the arthrodesis of critically unstable vertebrae in combination with the dynamic stabilization of adjacent moderately degenerated segments [34].

Three sequence test protocols for each spinal specimen were implemented to simulate the human spine under motion: (1) intact spine, (2) fusion at L4/5 with bilateral pedicle screws and titanium rods (static fixation only), and (3) supplementation of the L4/5 fusion with a dynamic component at L3/4 (hybrid fixation). Each test protocol had the same experimental procedures, which are described in Section 2.3. The loading modes included flexion, extension, and lateral bending.

**Figure 2:**
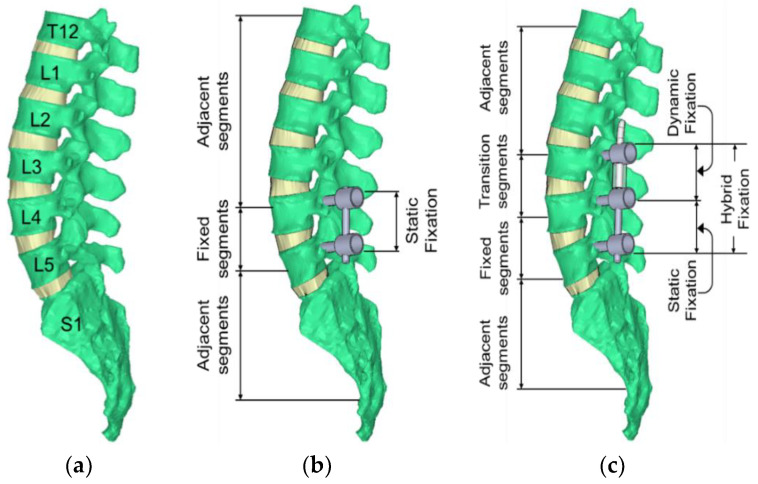
Depiction of spinal segments from T12 to sacrum (sagittal view): (**a**) intact specimen, (**b**) static fixation at L4/5 segments, and (**c**) hybrid fixation at L3–L5.

First, the initial testing was performed with each untreated (intact) spine specimen, before any surgical procedure, to obtain the baseline data. After the initial testing, each spine was instrumented posteriorly with a single-level static fixation at the L4/5 (static component was used stand-alone), and then the same biomechanical test procedure was performed to obtain the information of single-level fusion (static fixation). After the single-level fusion construct was tested, the hybrid fixation was then instrumented and tested. For the hybrid fixation, the specimen maintained the L4/5 rigid fixation. The dynamic fixation component was extended to the L3/4 level to form the hybrid fixation. According to the Dynesys implantation guide recommendations, the polyethene terephthalate (PET) tension cord was pre-tended to 300 N. In this study, an experienced orthopedic surgeon performed all spinal surgeries. Figure 2 depicts the intact specimen, L4/5 instrumented with static fixation, and a hybrid device (DTO) instrumented from L3 to L5 (dynamic fixation at L3/4 and static fixation at L4/5).

### 2.3. Biomechanical Test

**Figure 3:**
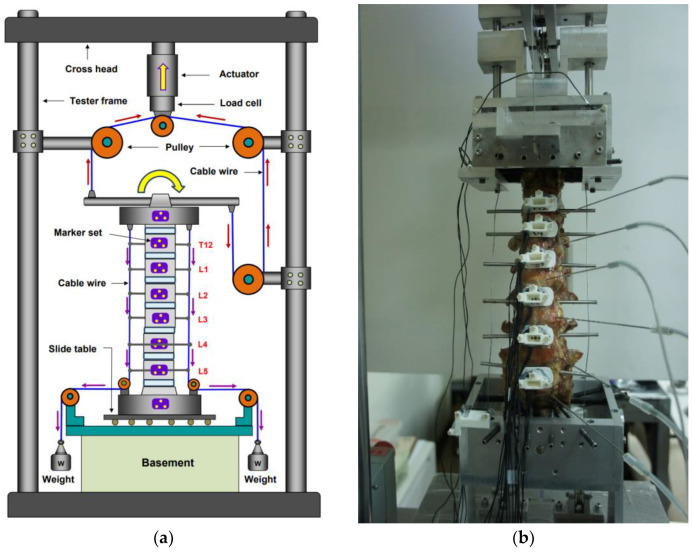
Schematic of the experimental setup: (**a**) a cable–pulley system was installed on the specimen to produce the pure moment, and the follower load was applied along the cable wires bilaterally by eyelets which were mounted laterally onto the vertebra; (**b**) Plexiglass marker sets were installed on the vertebra bodies (T12–L5).

A uniaxial electromechanic testing machine (Instron, Electro Pulse E3000, Norwood, MA, USA) was used to conduct all loading experiments. To produce a moment vector from the uniaxial tester, i.e., the stroke of the actuator of the tester, a cable–pulley mechanism was designed to carry out the pure moment in the test frame. A compressive follower load (FL = 500 N) from T12 to S1 was applied using a system of eyelets, cables, pulleys, and dead weights to simulate the body weight. Each eyelet passed approximately through the center of the vertebral body and was mounted laterally onto the vertebra; then, the load was applied bilaterally by cables and dead weights. Thus, the compressive load path can be applied following the curvature of the lumbar spine. The experimental setups are shown in Figure 3a. Custom-designed aluminum spinal fixtures were made to fix the specimen securely onto the testing machine. To mount the specimen onto the spinal fixture, eight embedded fixation screws were inserted into the cranial (T11) and caudal sacrum vertebra. A Plexiglass marker set containing three active light-emitting diodes was installed at the anterior aspects of each vertebra (T12–L5), these markers can be detected by an optoelectronic motion measurement system and applied to track the segmental motions.

The external forces were applied following three motion modes: flexion–extension (±7.5 Nm) and lateral bending (±7.5 Nm), with and without a follower load of 500 N [6,7,32,33,34,35,36]. Three cycles following a sinusoidal motion protocol of frequency 0.005 Hz were applied in each mode, with the third cycle data used for analysis [39,40,41,42,43,44]. During testing, the segmental motions (T12–L5) were evaluated using a 3D motion tracking system (Phoenix Technologies, Incorporated, Vancouver, Canada), and the kinematic data were recorded at a sampling rate of 10 Hz. Range of motion (ROM) measurements for each intervertebral level were obtained from the anteriorly placed vertebral tracking marker sets (three light-emitting diodes), as shown in Figure 3b. ROM was calculated as the range of the Euler angle corresponding to flexion, extension, and lateral bending modes of loading based on vertebral tracking body motion [39,41]. A local coordinate system for each vertebra was defined using three anatomical landmarks per body using the convention recommended by White and Panjabi [42] with the (+)x directed to the left, the (+)y directed superiorly, and the (+)z directed anteriorly. The needle pressure transducers (Catheter Tip Pressure Transducers, Gaeltec Devices Ltd., Dunvegan, UK) were inserted into the nucleus pulposus of the intervertebral disc to measure the IDP (or nucleus pressure, NP) while applying the external loads. The pressure data were recorded continuously during the testing. In each motion mode, the third cycle’s measured data were used for analysis. Baseline measurements of the ROM and IDP were performed for each intact spine in flexion/extension, right/left lateral bending, and with/without the compressive follower load. The change in IDP was evaluated for each motion with and without follower load.

### 2.4. Data and Statistical Analysis

To study the effects of both static fixation (one-level fusion) and hybrid fixation on the adjacent levels, the range of motion of the specimens with static/hybrid fixation was divided by the intact spine range of motion and was presented as the percentage of the intact specimen under the conditions of flexion, extension, and lateral bending combined with or without follower load. All results were presented as the mean and standard deviation (mean ± SD). Statistical comparisons were completed using a single-factor, repeated-measures analysis of variance. Tukey’s post hoc analysis was conducted to ascertain the statistically significant differences. Statistical significance was defined as a *p*-value < 0.05. All statistical analyses were performed using the statistical package software (SPSS 13.0 Inc., Chicago, IL, USA).

## 3. Results

### 3.1. Range of Motion (ROM)

**Figure 4:**
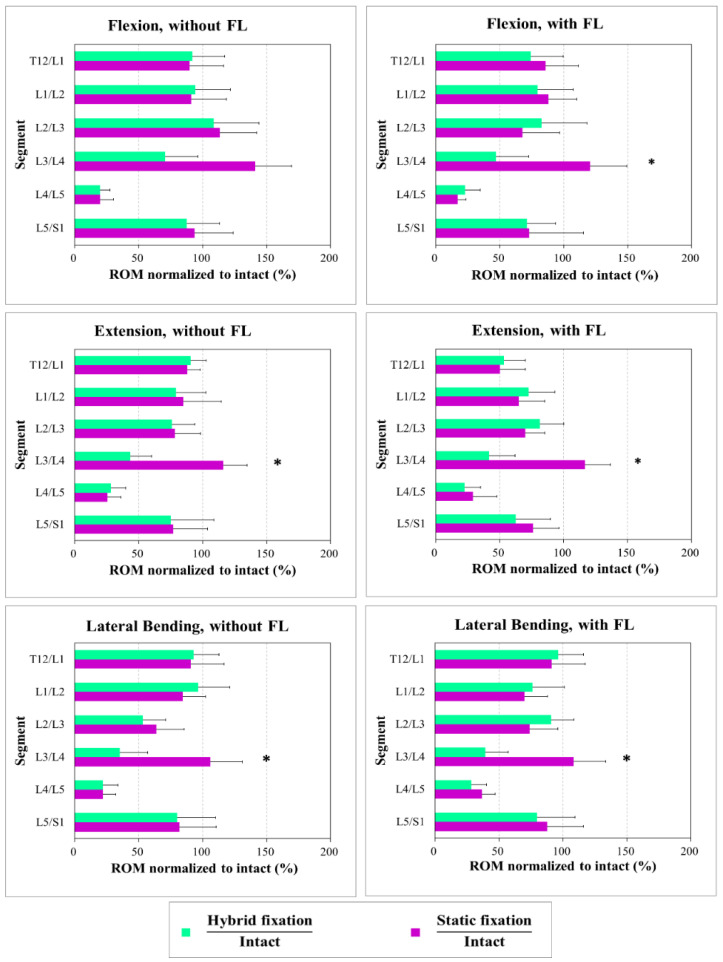
Percentage ROM of the specimens treated with static fixation and hybrid fixation to the intact spine under flexion, extension and lateral bending modes with and without a follower load. (*) Significance between hybrid and static fixations.

Figure 4 shows the percentage ROM for the specimens treated with static fixation and hybrid fixation to the intact spine under flexion, extension, and lateral bending modes with and without a follower load of 500 N. For all motion modes without follower loading, both the static constructs (static fixation at L4/5 only) and the hybrid constructs (static fixation at L4/5 and a dynamic component extended to L3/4) had significantly restricted motion at the L4/5 level. For the hybrid constructs, the ROM at the dynamic level (L3/4) was reduced in flexion at 71%, extension at 43%, and lateral bending at 35% compared with the intact results. Once the fusion level is performed, compensation may occur at the adjacent level, increasing the adjacent segmental motion. Our results show that the dynamic component of the DTO may reduce the ROM of the adjacent level. There was a significant difference in the ROM of L3/4 for the static constructs vs. hybrid constructs (flexion: 71% vs. 141% (*p* = 0.037); extension: 43% vs. 116% (*p* = 0.025); lateral bending: 35% vs. 106% (*p* = 0.042)). However, no significant changes were observed in other segments. Similar kinematic results were found in motion modes with a follower load of 500 N (flexion: 47% vs. 128% (*p* = 0.014); extension: 42% vs. 117% (*p* = 0.028); lateral bending: 40% vs. 109% (*p* = 0.039)).

### 3.2. Intradiscal Pressure (IDP)

**Figure 5:**
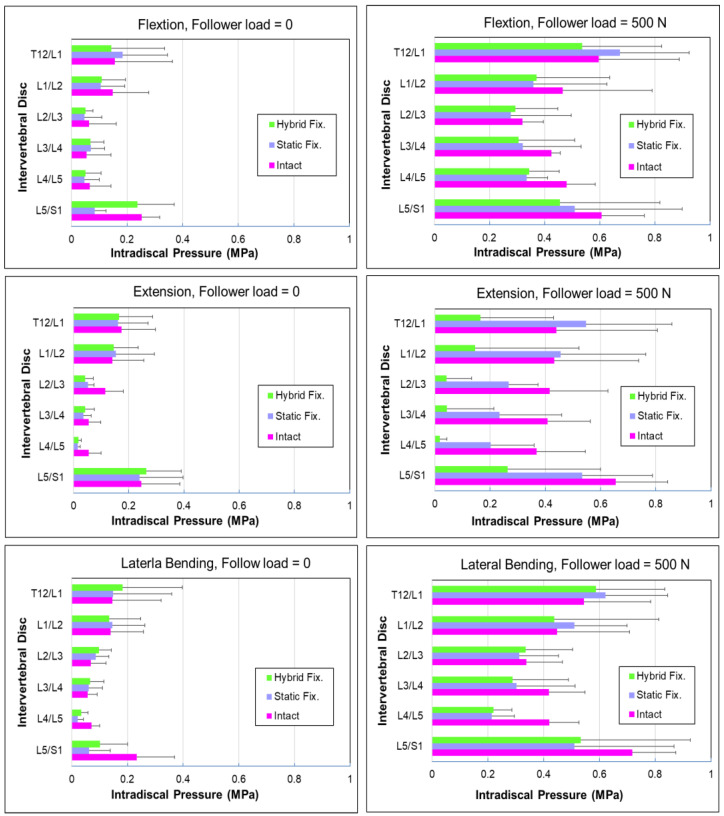
Values of intradiscal pressure (IDP) for each intervertebral disc segment.

Figure 5 shows the values of IDP for each intervertebral disc (IVD) segment in the lumbar spine. An in vitro biomechanical test was conducted to examine the effect of body weight on IDP at the spinal disc. A 500 N follower load was applied along the vertebral body to simulate the body weight with the pure moment of 7.5 N·m in flexion, extension, and lateral bending motion. It was found that the applied moment did not significantly increase the IDP but the follower load did. In this study, the measured IDP was less than 0.3 MPa during flexion, extension, and lateral bending motion without follower load. We think that the effect of different motion modes on the IDP was relatively minor; however, the IDP was markedly increased with follower loading. It was observed that the maximum IDP was 0.72 MPa at L5/S1 level under lateral bending combined with a 500 N follower load. Results indicated that the use of DTO restored IDP similar to that of the intact spine; thus, the increase in physiology loading could be an important factor for the increment in IDP.

Table 1 lists the change in intradiscal pressure (∆IDP) with 500 N follower load for intact, static fixation, and hybrid fixation constructs. The values of IDP were measured by the needle transducers, which were inserted into the center of each intervertebral disc (from T12/L1 to L5/S1). It was found that, as a 500 N follower load was applied, the ∆IDP values at L4/5 (static fixation alone) level were 0.087, 0.076, and 0.091 MPa for flexion, extension, and lateral bending posture, respectively. However, for the hybrid fixation, the ∆IDP values at L3/4 (dynamic fixation) level were 0.088, 0.069, and 0.070 MPa for flexion, extension, and lateral bending posture, respectively. At the L4/5 level (static fixation), the ∆IDP values were 0.093, 0.080, and 0.085 MPa for flexion, extension, and lateral bending posture, respectively.

## 4. Discussion

### 4.1. Bone Quality and Loosening of Fixations

Fatigue failure at the interface between the bone and screws, which frequently causes screw loosening, remains the biggest challenge for the dynamic stabilization system due to the need for continuous movement in such a motion-preservation device [33]. Clinical outcomes have indicated that the DTO provides a safe and effective alternative in the treatment of unstable lumbar spinal diseases, with a reported satisfaction rate ranging from 60% to 90% [24,25,26]; however, complications including adjacent segment diseases, screw loosening, and screw breakage have been reported in the postoperative period [28,29,30,31,32]. Research showed that BMD was not correlated with screw loosening in the dynamic stabilization system. The incidences of screw loosening in Dynesys dynamic stabilization were approximately 5% per screw and 20% per patient in 2–5 years post operation [28,34]. Wu et al. [44] reported that screw loosening in dynamic stabilization is not uncommon (4.7% of screws in 19.8% of patients), with older patients or those with diabetes having higher rates of screw loosening. Screw loosening can be asymptomatic, with a chance of osseous integration on later follow-up.

In this study, the BMD values showed that the specimens were osteoporosis-free, and no screw loosening or screw breakage was found in any specimen. We think that the stiffness of the dynamic component of DTO is less than that of a rigid rod, while the dynamic component is designed to shift rather than bear the entire loading of the lumbar spine. However, durability remains the most frequent concern of the non-fusion constructs, especially in patients with inadequate BMD. The effect of cyclic loading on the pull-out strength of the Dynesys screw in vertebrae remains elusive. Further investigations on the complications and functional outcomes of patients with osteoporosis who receive a pedicle screw-based posterior dynamic stabilization system (DTO) are required.

### 4.2. Range of Motion at the Implanted and Adjacent Levels

The motion segment is the traditional unit of study in spinal kinematics. It constitutes two adjacent vertebrae and the intervertebral disc. The range of motion is described in terms relative to the subjacent vertebra; a larger motion of the vertebra body leads to greater disc stresses. When the bending loads are applied during flexion, extension, or lateral bending, the spine is subjected to tension on its convex side and compression on its concave side. On the compression side, the disc bulges, while it contracts on the tension side. Thus, the bending loads can be thought of as a combination of tensile and compressive loads [41]. Experiments have confirmed that the stresses in the disc due to pure compression load are not large enough to cause disc failure; however, the risk of disc failure is greater with tensile loading as compared with compressive loading [45,46,47,48].

In this study, the segmental motion of the hybrid construct causes a greater overall increase in adjacent level effects than the static construct in all the loading modes (i.e., flexion/extension and lateral bending, with/without a follower load). In assessing the effect of each construct separately on the adjacent levels for flexion/extension, we found that the static construct caused a significant increase in adjacent-level effects to its nearest cranial adjacent levels (L3/4). However, there was no significant effect on the supra-adjacent levels (L1/2 and L2/3) in flexion and extension. This is because the static fixation at L4/L5 caused stress concentration and a significant motion change at L3/4; the compensative phenomenon of lumber motion after L4/5 fusion mainly focused on the L3/4 level, enlarging the segmental motion of L3/4; the dynamic component decreased the compensation of L3/4 level.

### 4.3. Effects of Follower Load on the Intradiscal Pressure (IDP)

The follower load employed in this study simulated the physiological loading increase in the human spinal disc. Under the extension, when a 500 N follower load was applied, the IDP increased by 0.069 MPa and 0.080 MPa at L3/4 and L4/5 levels, respectively. These values are lower than other ∆IDP values in Table 1. This is because the extension motion increased the lordosis of the lumbar spine, and the DTO core pretension was carried at the posterior of the spine, enhancing the cantilever support of the pedicle screw, increasing the resistance of vertebral loads, and then decreasing the compressive stress of the disc. Schmoelz et al. [46] investigated the load transfer to the intervertebral disc stabilized with either rigid instrumentation or Dynesys dynamic stabilization system. In their study, IDP was measured using flexible pressure transducers. Their results showed that, in extension, dynamic and rigid stabilization significantly reduced IDP, whereas no significant difference was observed in flexion. The pressure increases in flexion and decreases in extension could be related to the posterior shift of the axis of rotation caused by the application of posterior instrumentation. For lateral bending, no significant reduction of IDP was found.

In our study, both intact and fusion levels exhibited these trends at proximal and distal adjacent segments relating to the spine without follower load. In the L4/5 static fixation model, the ∆IDP of the nucleus pulposus at the adjacent segments (L3/4) was higher than that of the corresponding segment of the intact model under three different motion directions. Increased IDP enhanced tensile stress on the annulus fibers, which led to excessive stress on the intervertebral disc and stimulated disc degeneration, particularly in the nucleus pulposus [47]. Clinical and biomechanical studies have shown that fusion surgery can increase the biomechanical stress at the proximal and distal adjacent segments, and the proximal adjacent segment was more vulnerable than the distal adjacent segment [42]. The stress concentration at the adjacent segments following fusion surgery could potentially increase the risk of disc degeneration. Although many papers reported that the patient’s age, multiple-level fusion [48,49,50,51,52], sagittal malalignment, posterior interbody fusion, and pre-existing disc degeneration [53,54,55] could be risk factors for adjacent segmental disease, others contradicted these risk factors. Kumar et al. [52] reported that gender, different types of fusion, and fusion level were not risk factors for ADS. Stress on the disc includes the IDP and the annulus fibrosis stress; although IDP can be experimentally measured by needle pressure transducers, the annulus disc stress is difficult to evaluate during testing. Several finite element studies attempted to theoretically analyze the annulus fibrosis stress under spinal motion; however, the values of this stress were not validated. The annulus fibrosis stress at intervertebral discs at the lumber spine may lead to early intervertebral disc damage. We believe the progression of adjacent segmental diseases following lumbar spine fusion is multifactorial, requiring additional research to identify and quantify the contributing risk factors.

### 4.4. Effects of Stiffness and Cord Tension of DTO

The biomechanical characteristics of Dynesys have been evaluated by several laboratories using in vitro experimental tests or finite element (FE) analysis. Liu et al. investigated the effect of the cord pretension of the Dynesys dynamic stabilization system on the biomechanics of the lumbar spine using a finite element method. Their results showed that increasing the cord tension of the Dynesys dynamic stabilization system from 100 to 300 N resulted in a further ROM decrease by 15% in flexion and an ROM increase by 17% in extension [53]. The authors also indicated that adjusting core pretension might affect ROM, facet contact force, and annulus stress within the construct but not in the adjacent segment, particularly in flexion/extension. Theoretically, increasing cord pretension causes an increase in stiffness under flexion and a slight reduction in stiffness under extension at the implanted level when compared with the intact lumbar spine. A higher stiffness decreased the segmental motion; in contrast, a lower stiffness (flexible) led to greater ROM.

Some clinical studies have reported the long-term complications of adjacent segmental diseases [24,25,32,53], which may be explained by prominently increased disc annulus stress of the adjacent levels under flexion following the implantation of the Dynesys dynamic stabilization system when using higher cord pretension, whereas a lower cord pretension could reduce the possibility of developing adjacent segmental diseases [54,55]. However, more evidence is needed to support this assumption. In vitro and in vivo studies have confirmed the time-dependent deformation of spine segments under stress [56,57,58,59,60,61,62]. In these studies, short-term viscosity, with intervals of several hours, was examined, but the long-term effects of viscosity on spinal structures have yet to be appropriately studied. Their experiments did not consider the viscoelastic behaviors of the PCU spacer and PET tension cord.

In our study, a 300 N preload was applied to the PET cords to distract the disc during implantation. The magnitude of pretension was according to the Dynesys implantation guide recommendations. After the preload was applied to the dynamic component of the DTO system, the PET cord was pre-stretched up to 300 N and fixed with the Dynesys screw using a set screw, and then the PCU spacer held the PET cord at a constant length, and the cord tension gradually lost a part of its tension due to relaxation. Additionally, the spinal discs behaved with a creep response under an external load, and the creep response led to shortening at the implanted level and then relaxed the pretension of the cord. A different cord pretension may change the stiffness of the Dynesys system and result in diverse clinical outcomes. The overall medium- and/or long-term effect of the DTO system due to the relaxation of core tension remains unknown. Mageswaran et al. conducted a biomechanical study which compared the fusion and hybrid constructs. Their results showed that the hybrid stabilization system had similar characteristics to the fusion construct because of greater stress in adjacent segments in the hybrid construct [32]. The finite element study also revealed stiffness resulting from the Dynesys system, which is the same as rigid fixation [61]. However, Durani et al. reported a contrasting result. Their study showed that the dynamic stabilization system could reduce the hypermobility caused by extended arthrodesis [62]. In addition, the authors indicated that the IDP at the segment adjacent to the fusion was reduced when a dynamic stabilization system was added above the segment after fusion. They concluded that hybrid surgery might have a possible preventative effect on degenerative disc changes at the adjacent segment.

### 4.5. Study Limitations

This study had several limitations. Firstly, the implantation of the DTO spacer requires cutting the PCU spacer to match the native distance of the pedicles, as well as the location and angle of the pedicle screw insertion. Niosi et al. demonstrated that space length directly influences lordosis, segmental motion, and loading [63]. In the current study, the spacer length varied bilaterally in each specimen and between specimens, all spacers were cut to fit the distance between L3 and L4 segments, and the variation in spacer length existed in all treatment conditions. Secondly, the cadavers lacked the physical reality of living human patients. In reality, the direction and magnitude of the lumbar muscle forces are constantly changing and responding to the stability requirements of the spine. Although the path of the follower load was applied along the curvature of the spine, the static (constant) follower load used in our test was under a simulated biomechanical condition, not in a physiologic condition. Thirdly, the state of degeneration of the disc and facet degeneration at the level adjacent to the implantation could have affected the biomechanical behaviors of the hybrid dynamic stabilization system. The influence of intrinsic disc degeneration, neutral zone, and stiffness of the implant and disc are not reported here. Fourthly, although this study used the human spine, recovery behaviors of the lumbar units were not considered. Lastly, the number of specimens was small, and the axial torsional mode was not included in our study; the intervertebral ROM and IDP under flexion, extension, and lateral bending modes were the primary study metrics.

## 5. Conclusions

A significant increase in range of motion can be observed at the adjacent segments to the interbody fusion. The hybrid stabilization system’s dynamic component protects the adjacent level from excessive motion. The follower load has a significant effect on the IDP. However, the present study cannot support the safety of dynamic hybrid devices in those cases if the reduction in adjacent segmental diseases is the main target. Due to the small number of specimens in the present study, the determination of the medium- or long-term effects of hybrid dynamic stabilization and its impact on the adjacent segment requires further biomechanical studies and clinical research to optimize the implant design to decrease the complications and improve patient safety.

## Figures and Tables

**Table 1:** Change in intradiscal pressure (∆IDP) after 500 N follower load was applied for intact, static, and hybrid fixation constructs at flexion, extension, and lateral bending.

Discal Level	Intact Without Implanted ∆IDP (MPa)			Fusion L4/5 (Static) ∆IDP (MPa)			Hybrid L3/4 (Dynamic) + L4/5 (Static) ∆IDP (MPa)		
	Flexion	Extension	Lateral Bending	Flexion	Extension	Lateral Bending	Flexion	Extension	Lateral Bending
T12/L1	0.440	0.266	0.399	0.490	0.385	0.474	0.393	0.383	0.405
L1/2	0.318	0.291	0.309	0.253	0.301	0.365	0.262	0.287	0.304
L2/3	0.256	0.301	0.270	0.230	0.266	0.226	0.243	0.232	0.237
L3/4	0.371	0.352	0.361	0.251	0.292	0.302	0.088	0.069	0.070
L4/5	0.405	0.383	0.370	0.087	0.076	0.091	0.093	0.080	0.085
L5/S1	0.394	0.409	0.429	0.425	0.333	0.448	0.219	0.299	0.432

## Data Availability

Data are contained within the article.
